# Evaluation of different combination of pam2CSK4, poly (I:C) and imiquimod enhance immune responses to H9N2 avian influenza antigen in dendritic cells and duck

**DOI:** 10.1371/journal.pone.0271746

**Published:** 2022-07-19

**Authors:** Aiguo Zhang, Deyin Li, Chao Song, Huiyuan Jing, Hongfei Li, Junxian Mi, Guizhi Zhang, Shuangxing Jin, Xiaoli Ren, Heping Huangfu, Dongmei Shi, Ruiai Chen

**Affiliations:** 1 College of Veterinary Medicine, Henan University of Animal Husbandry and Economy, Zhengzhou, Henan, China; 2 Huannong (Zhaoqing) Institute of Biotechnology Co. Ltd., Zhaoqing, Guangdong, China; 3 Henan Poultry Disease Prevention and Control Engineering Technology Research Center, Zhengzhou, Henan, China; 4 College of Animal Science and Technology, Henan University of Animal Husbandry and Economy, Zhengzhou, Henan, China; 5 College of Veterinary Medicine, South China Agricultural University, Tianhe District, Guangzhou, Guangdong, China; 6 Zhaoqing Branch Center of Guangdong Laboratory for Lingnan Modern Agricultural Science and Technology, Zhaoqing, Guangdong, China; University of South Dakota, UNITED STATES

## Abstract

Current commercial H9 avian influenza viruses (AIVs) vaccines cannot provide satisfactory antibody titers and protective immunity against AIVs in duck. Toll like receptors (TLR) ligand as AIVs adjuvants can activate dendritic cells to improve immune responses in multiple animals, while the studies were absent in duck. Therefore, we investigated TLR ligands pam2CSK4, poly (I:C) and/or imiquimod enhance immune responses to inactivated H9N2 avian influenza antigen (H9N2 IAIV) in peripheral blood monocyte-derived dendritic cells (MoDCs) and duck. *In vitro*, we observed that transcription factor NF-κB, Th1/Th2 type cytokines (IFN-γ, IL-2 and IL-6) and the ability of catching H9N2 IAIV antigen were significantly up-regulated when H9N2 IAIV along with TLR ligands (pam2CSK4, poly (I:C) and imiquimod, alone or combination) in duck MoDCs. Also, the best enhancement effects were showed in combination of pam2CSK4, poly (I:C) and imiquimod group, whereas IFN-α showed no significant enhancement in all experimental groups. *In vivo*, the results demonstrated that the percentages of CD4^+^/ CD8^+^ T lymphocytes, the levels of Th1/Th2 type cytokines and H9N2 HI titers were significant enhanced in combination of pam2CSK4, poly (I:C) and imiquimod group. However, pam2CSK4 alone or combining with imiquimod showed no enhancement or additive effects on Th1 cytokines (IFN-γ and IL-2), Th2 cytokines (IL-6) and HI titers in Muscovy duck, respectively. Taken together, our results concluded that not all TLR ligands showed enhancement of immune responses to H9N2 IAIV in duck. The combination of poly (I:C), imiquimod and pam2CSK4 that can be an effectively adjuvant candidate for H9N2 AIVs inactivated vaccine in duck, which provide novel insights in explore waterfowl vaccine.

## Introduction

Because of duck infected with H9N2 avian influenza viruses (AIVs) are generally overlooked due to the lack of clinical symptoms. Since 1994, H9N2 virus was first isolated in chicken in China, H9N2 subtype quickly spread to most areas of China and replaced H5N6 and H7N9 as the dominant AIVs subtype in duck in recent year, moreover, it is considered to be a potential candidate for a future pandemic [1–3]. The strategy recommended to control or eradicate H9N2 AIVs is using vaccine. However, duck receiving an inactivated H9N2 AIVs vaccine showed lower antibody titers than chicken and escaped from protection of the present inactivated vaccine due to cannot induce sufficient cellular immunity, resulted in H9N2 AIVs continue to circulate in several countries, which make major economic losses to the poultry industry and threaten public health [1,4–6].

Toll like receptors (TLR) play crucial roles in humoral and cellular adaptive immune responses [7–9]. After TLRs bind to the corresponding TLRs ligands, the TLRs signaling pathway can be activated. TLR2 and TLR7 can be transduced through MyD88-dependent signaling pathway [10]. TLR3 can be transduced through the TRIF signaling pathway. Though TRIF and MyD88-dependent signaling pathways involve different players, they share some common regulators such as NF-κB. Furthermore, the different signaling pathways also have the synergistic effects under a certain conditions [11]. NF-κB is a major transcription factor in TLR signaling pathway, which can cause the secretion of various immune-related cytokines, thereby activating the body’s immune response [10]. TLRs are differentially expressed in immune cells, and preferentially expressed in antigen-presenting cells (APCs) such as dendritic cells (DCs) [10,12]. DCs serve as a passage between innate and adaptive immune response which has an irreplaceable role in the modulation of host immunity [12]. TLR ligands can activate DCs effectively, thereby enhancing the capacity of phagocytosis and antigen presenting of DCs, and make T cells activation and regulation, induced the enhancing of humoral and cellular immune response capability [12,13].

Previous studies have shown that some TLRs ligands, such as TLR2 ligand pam2CSK4, TLR3 ligand poly (I:C) and TLR7 ligand imiquimod have been explored as potential adjuvant for the influenza vaccine in chicken, mice and so on, respectively [9,14–16]. Although most of the TLRs and signaling pathways are similarly between chicken to duck, differences are still evident between these two avian species. For example, TLR21 is absent in duck yet present in chicken. The TLR3 expression in duodenum is lower in duck yet higher in chicken [13,17,18]. Furthermore, a combination of TLR ligands might minimize the dose and side effect, mimic the natural infection and induce more balanced or desirable immune responses compare to a single TLR ligand [16,19,20]. Importantly, DCs play a critical role in the innate and adaptive immune system in the host. However, there is limited information on the effects of DCs with multiple TLR ligands *in vitro* and the use of TLR ligands combinations to enhance antibody responses *in vivo* for AIVs in duck.

Hence, in the present study, we aimed to evaluate the immunomodulatory effect and mechanism of TLR2 ligand pam2CSK4, TLR3 ligand poly (I:C) and TLR7 ligand imiquimod, alone or in combination, to inactivated H9N2 avian influenza antigen *in vitro* of dendritic cells and *in vivo* of Muscovy duck. Our experiments may provide novel insights to further explore in AIVs inactivated vaccine development in duck.

## Materials and methods

### Virus, TLR ligands and experimental birds

H9N2 AIVs (A/Chicken/Jiangsu/DHN12/2013 (H9N2, HA clade: 4.2.5)) was obtained from the Guangdong Enterprise Key Laboratory of Biotechnology R&D of Veterinary Biologics. Briefly, the H9N2 vaccine candidate virus (50% egg infective dose, EID_50_ at 10^8^/0.1 mL) was propagated in 10-day-old embryonated SPF chicken eggs (Guangdong Enterprise Key Laboratory of Biotechnology R&D of Veterinary Biologics). Virus titers were measured following the method of Reed & Muench as described previously [6,21]. Virulent H9N2 AIVs was inactivated with 0.05% formaldehyde (Ferak Berlin Gmbh, Berlin, Germany) by incubating at 4°C for 12 h. Complete inactivation of the virus was confirmed through repeated passages in 10-day-old embryonated SPF chicken eggs and Madin-Darby canine kidney cells as previously described [6,21]. The inactivated H9N2 AIVs antigen (H9N2 IAIV) was purified on a discontinuous sucrose density gradient as described [22].

The 1-day-old white Muscovy (Wens Foodstuff Group, China) was raised in an isolator to allow the animals to eat and drink normally. These ducks were raised for two weeks to adapt to their living environment. All duck serum samples were confirmed to be free of any AIV infection in the study. Adjuvant of TLR2 ligand pam2CSK4, TLR3 ligand poly (I:C) and TLR7 ligand imiquimod were purchased from InvivoGen (San Diego, CA).

### Quantitative real-time PCR

The total RNA of test samples were extracted and reversely transcribed to cDNA as previously study described [6]. The reaction mixture contained a volume of 20 μL with 10 μL of SYBR Premix Ex TaqTM II (Tli RNaseH Plus, TaKaRa, Japan), 2 μL of cDNA and 0.4 mM primers. Quantitative real-time PCR (qPCR) was performed by Rotor Gene Q real-time detection system (QIAGENGmbH, Manheim, Germany). The abundance of individual mRNA transcript in each sample was assayed three times and normalized to that of β-Actin mRNA (as an internal control). Relative transcript levels were quantified by the 2^−ΔΔCt^ (where Ct is threshold cycle) method and shown as fold changes relative to the level for the control group. The amplification program was 95°C for 30 s, followed by 40 cycles with 5 s at 95°C for denaturation, 30 s at 55°C for annealing, and 30 s at 72°C for extension. After the amplification phase, melting curves were routinely performed to confirm the presence of a single PCR product.

### Experiment 1. Evaluation of TLR ligands induced immune response to H9N2 IAIV on duck peripheral blood monocyte-derived dendritic cells (MoDCs)

#### Preparation of duck MoDCs

Duck MoDCs were induced and differentiated from peripheral blood monocytes (PBMCs) of healthy duck. In brief, the peripheral blood was collected from 2-week old healthy ducks (n = 3) by sterile vessels containing EDTA anticoagulant. PBMCs were isolated according to the manufacturer’s instructions of chicken peripheral blood lymphocyte isolation kit (TBD, China). Subsequently, PBMCs were cultured in RPMI-1640 medium containing 10% FBS, 100 IU/mL penicillin, 100 μg/mL streptomycin and stimulated with 20 ng/mL recombinant chicken IL-4 (Innovative Research of America, USA), 40 ng/mL recombinant chicken GM-CSF (Innovative Research of America, USA) at a density of 1× 10^7^/mL in six-well plates at 37°C with 5% CO_2_ for 7 days to make cells differentiate into MoDCs. Half of the culture medium was removed with the replacement by equal volume of fresh medium every three days. 7 days later, most of cells showing a single or clustered non-adherent, irregular shape, veiled and/or dendrites (S1 Fig). In addition, PE-mouse anti-chicken MHC-II antibody (Southern Biotech, USA) was used to confirm the percentages of cells expressing MHC-II by flow cytometry for evaluating the differentiation, which reached 66.5% at 7 days (S2 Fig).

#### Experimental design and immune response genes test *in vitro*

Duck MoDCs were divided into eight groups: Group a: pam2CSK4-H9N2 IAIV group, Group b: poly(I:C)-H9N2 IAIV group, Group c: imiquimod-H9N2 IAIV group, Group d: pam2CSK4-imiquimod-H9N2 IAIV group, Group e: poly (I:C)-imiquimod-H9N2 IAIV group, Group f: pam2CSK4-poly (I:C)-imiquimod-H9N2 IAIV group, Group g: H9N2 IAIV group and Group h: RPMI-1640 group. For groups a–f: MoDCs were treated with pam2CSK4 (10 μg/mL), poly (I:C) (20 μg/mL) and/or imiquimod (5 μg/mL) along with 1× 10^7^ EID_50_ H9N2 IAIV per well, respectively. For group g: MoDCs were treated with 1× 10^7^ EID_50_ H9N2 IAIV alone per well. For group h: MoDCs were cultured with RPMI-1640 and used as mock control. Duck MoDCs were harvested at 6 and 12 h post-stimulation (n = 3/group/time point) to determine the relative mRNA expressions of TLR signaling pathway key node proteins (MyD88, TRIF and NF-κB), Th1-type cytokines (IL-2 and IFN-γ), Th2-type cytokines (IL-6), proinflammatory cytokines (IFN-α and TNF-α) and cell surface molecules (MHC-I and MHC-II). Quantitative real-time PCR primers used in this study were listed in Table 1.

**Table 1:** Primers used in quantitative real-time PCR.

Target gene	Sequence (5’~3’)	GenBank no.
IL-2-F	GACTACAGCTTATGGAGCACCTCT	AF294323
IL-2-R	ACTCCTTTGTGTCATTTGGTGTGT	AF294323
IL-6-F	CCCAGAAATCCCTCCTCACA	JQ728554.1
IL-6-R	CAAATAGCGAACAGCCCTCAC	JQ728554.1
CD4-F	GTCCCATCCCACCTAATGTCC	NM001310403.1
CD4-R	CTCCACCTTTGTTTCACTCTGTTTT	NM001310403.1
CD8α-F	GAAGTCCTTCAAGGCAGAG	JX051841
CD8α-R	AGACGTCCCTCTTGGTGAC	JX051841
MHC-I-F	GAAGAGCAAGCAGGGGAAGA	AB115246.1
MHC-I-R	TGAAGCAGAGCGGTTAGACAC	AB115246.1
MHC-II-F	CTCGAGGTCATGATCAGCAA	AY905540.1
MHC-II-R	TGTAAACGTCTCCCCTTTGG	AY905540.1
MYD88-F	TCCATCAGCGGAGAGCTTAT	NM001310832.1
MYD88-R	CTTCATGGCTTTGCACTTCA	NM001310832.1
TRIF-F	GCTTTCAGGATGCTTTGGAG	KJ466051.1
TRIF-R	CTTGGGAACAAAAGGGATGA	KJ466051.1
NF-κb-F	TCAACGCAGGACCTAAAGACAT	XM013097692
NF-κb-R	GCAGATAGCCAAGTTCAGGATG	XM013097692
IFN-α-F	TCCTCCAACACCTCTTCGAC	DQ861429
IFN-α-R	GGGCTGTAGGTGTGGTTCTG	DQ861429
IFN-γ-F	TCAGAGACCTCGTGGAACTGTC	AJ012254
IFN-γ-R	ACTGGCTCCTTTTCCTTTTGG	AJ012254
TNF-α-F	ACAGGACAGCCTATGCCAAC	NM001310341
TNF-α-R	ACAGGAAGGGCAACACATCT	NM001310341
BAFF-F	GTGCCCCTGTTTCTTCCTTC	DQ445092.1
BAFF-R	CCTGTTTCTGCTCCCGTTC	DQ445092.1
β-Actin-F	TGATGGACTCTGGTGATGGTG	EF667345.1
β-Actin-R	ATTTCTCTCTCGGCTGTGGTG	EF667345.1

#### Flow cytometry analysis in MoDCs

The MoDCs were harvested at 12 h post-stimulation (n = 3/group) to determine the function of MoDCs phagocytose H9N2 IAIV. In brief, MoDCs were fixed and permeability using Cytofix/Cytoperm according to the manufacturer’s instructions (BD Biosciences, San Jose, USA). MoDCs were then stained with Rabbit FITC-H9N2 HA protein monoclonal antibody (Sino Biological Inc. Beijing, China) at 4°C for 1 h. Then washed twice with PBS, and re-suspended in 4% paraformaldehyde for flow cytometry (FACSAria III, BD Biosciences). The data were analyzed using the Flowjo7.6 software.

### Experiment 2. Evaluation of immunomodulatory activity of pam3CSK4 and/or poly(I:C) and/or imiquimod administered with H9N2 IAIV in duck

#### Experimental design in vivo and haemagglutination inhibition test

Two-week-old white Muscovy duck (n = 40) were allotted randomly to one of eight groups (n = 5/group) as showed in Table 2. All groups of duck were intraperitonealed by H9N2 AIV vaccine or PBS twice at a 10-day interval. At 21 days post-vaccination (dpv), all ducks were euthanized and the sera were harvested for the detection of haemagglutination inhibition test (HI) [6]. At the same time, spleen tissues (n = 3/group) were collected from each group. One part of splenic lymphocytes was used for detection the percentages of H9N2 AIV-specific CD4^+^ and CD8^+^ T lymphocytes, while the other part of spleen tissue was stored in liquid nitrogen for detecting cytokines mRNA expression.

**Table 2:** Immunization plan in the experimental ducks.

Group	H9N2 IAIV (2× 10^7.0^ EID_50_/duck)	Pam3CSK4 (10 μg/duck)	Poly(I:C) (20 μg/duck)	Imiquimod (5 μg/duck)
A	+	+	-	-
B	+	-	+	-
C	+	-	-	+
D	+	+	-	+
E	+	-	+	+
F	+	+	+	+
G	+	-	-	-
H (PBS control)	-	-	-	-

#### Flow cytometry analysis in splenic lymphocytes

In order to evaluate the percentages of CD4^+^ T and CD8^+^ T cells in splenic lymphocytes of duck. The duck spleen lymphocyte was separated and then incubated with mouse anti-duck CD4 or CD8 antibody (Bio-Rad, USA) 1:100 at 4°C for 1 h. After washing twice with washing liquid, FITC-Sheep anti-mouse/rat IgG (H+L) (Abcam, Cambridge, UK) secondary antibody 1:1000 was added and incubated in darkness for 1 h. Then washed twice and re-suspended in 4% paraformaldehyde. The expressions of different cell surface markers were analyzed by flow cytometry.

#### Evaluation of cellular immune response in duck

TLR ligands induced cytokines mRNA expression in spleen tissue was evaluated at 21 dpv using qPCR to determine T cell function in the experimental ducks. In brief, isolation of spleen totals RNA and synthesis of cDNA according to the manufacturer’s instructions. Expression levels of mRNA of cytokines (IL-2, IFN-α, IFN-γ, IL-6 and TNF-α) and immune-associated genes (MHC-I, MHC-II, CD4, CD8 and BAFF) were analyzed by qPCR. Quantitative real-time PCR primers used were listed in Table 1.

### Statistical analysis

Statistical analysis was performed used using GraphPad Prism 6 software (GraphPad Software, CA, USA). Data are shown graphically as the geometric mean of the fold change plus the standard error of the mean (SEM). Differences in the expression level of immune-related genes in duck MoDCs and spleen tissues were analyzed using two-way ANOVA followed by Bonferroni’s test. Data from the HI levels and flow cytometry analysis were analyzed by one-way ANOVA with Tukey’s post-hoc test. The results were considered statistically significant when *P*-values were less than 0.05.

#### Ethics statement

All animal experiments were carried out in strict accordance with the guidance of both of the Centers for Disease Control and Prevention’s Institutional Animal Care and Use Committee and the Association for Assessment and Accreditation of Laboratory Animal Care International. The protocol was approved by the Committee on the Ethics of Animal Experiments of Animal Biosafety Level 3 Committee of South China Agricultural University. Surgery and euthanasia was performed under anesthesia with sodium pentobarbital solution (100 mg/kg body weight) via intravenous route to minimize suffering.

## Results

### Activation of TRIF/MyD88-NF-κB signaling pathway in duck MoDCs treated with H9N2 IAIV along with combination of pam2CSK4, poly (I:C) and/or imiquimod

**Fig 1:**
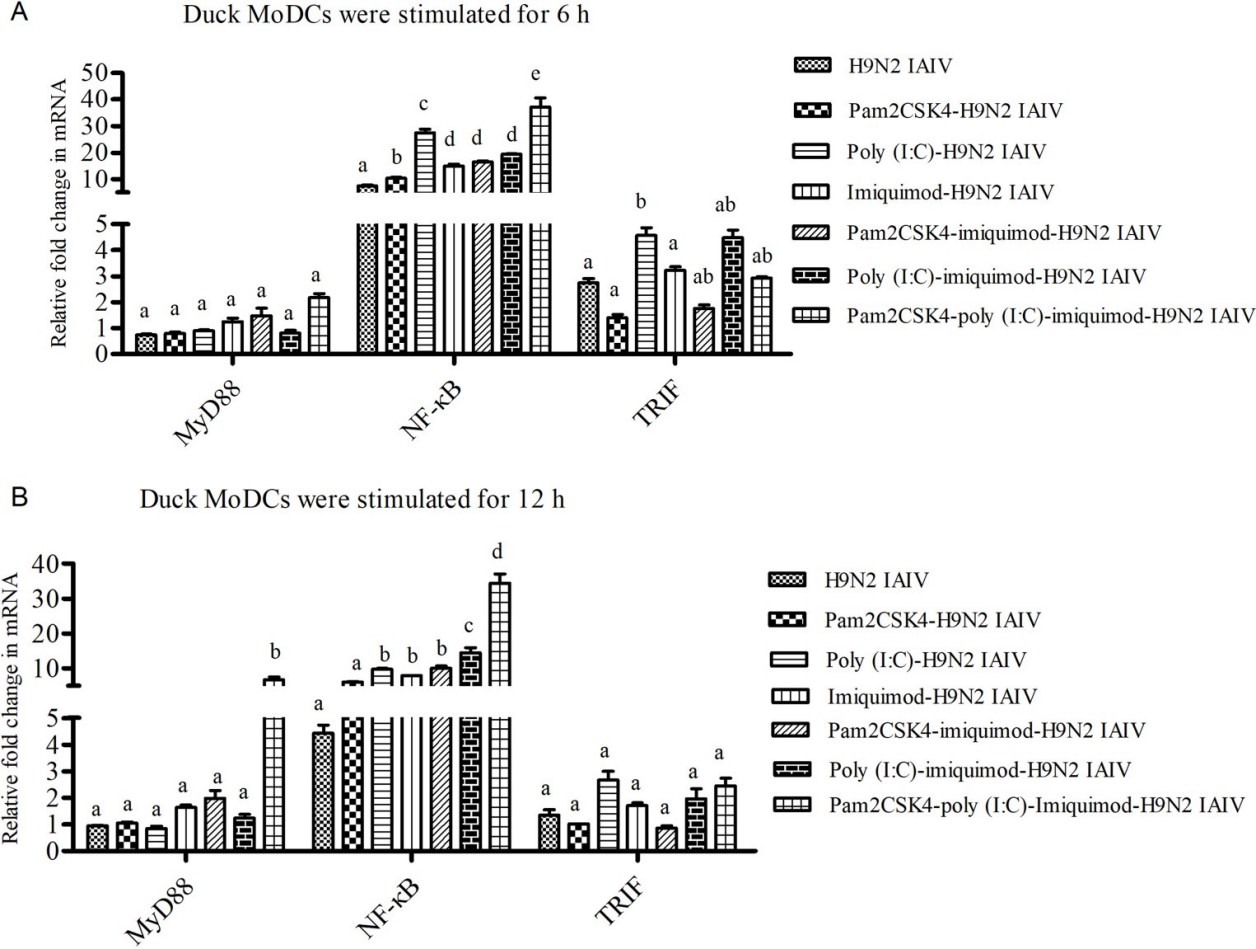
The mRNA levels of MyD88, TRIF and NF-κB in duck MoDCs stimulated with TLR ligand (s) and H9N2 IAIV at 6 h and 12 h. Then mRNA levels of MyD88, TRIF and NF-κB were detected at 6 h (A) and 12 h (B) post-stimulated via qPCR. Relative mRNA levels are shown as fold changes relative to the level for the control cells of the RPMI-1640 group. Expression of target genes was normalized to those of duck β-Actin. Data were indicated as mean ±SEM. Different letters (a~e) mean significant difference (*P*<0.05). If there is one same letter, for example, ab means has no significant difference between adjacent a or b (*P*>0.05).

The mRNA levels of MyD88, TRIF and NF-κB were measured in duck MoDCs which stimulated with pam2CSK4, poly (I:C) and/or imiquimod along with H9N2 IAIV for 6 h and12 h by qPCR. In H9N2 IAIV, pam2CSK4-H9N2 IAIV and pam2CSK4-poly (I:C)-H9N2 IAIV groups, the mRNA levels of TRIF and MyD88 remained at the basal level (Fig 1).The mRNA level of TRIF was higher in poly (I:C)-H9N2 IAIV group at 6 h, and MyD88 was higher in pam2CSK4-poly (I:C)-imiquimod-H9N2 IAIV group at 12 h (Fig 1).The mRNA level of NF-κB was significantly elevated in in all TLR ligand(s)-H9N2 IAIV groups, and the highest level showed in pam2CSK4-poly (I:C)-imiquimod-H9N2 IAIV group compared with other groups at 12 h post stimulation(Fig 1). Notably, the mRNA levels of NF-κB showed no difference between poly (I:C)-H9N2 IAIV and pam2CSK4-poly (I:C)-H9N2 IAIV groups, but higher shown in poly (I:C)-imiquimod-H9N2 IAIV than imiquimod-H9N2 IAIV at 12 h post stimulation (Fig 1).

### Combination of pam2CSK4, poly (I:C) and/or imiquimod with H9N2 IAIV enhances duck MoDCs activation and cytokine production

**Fig 2:**
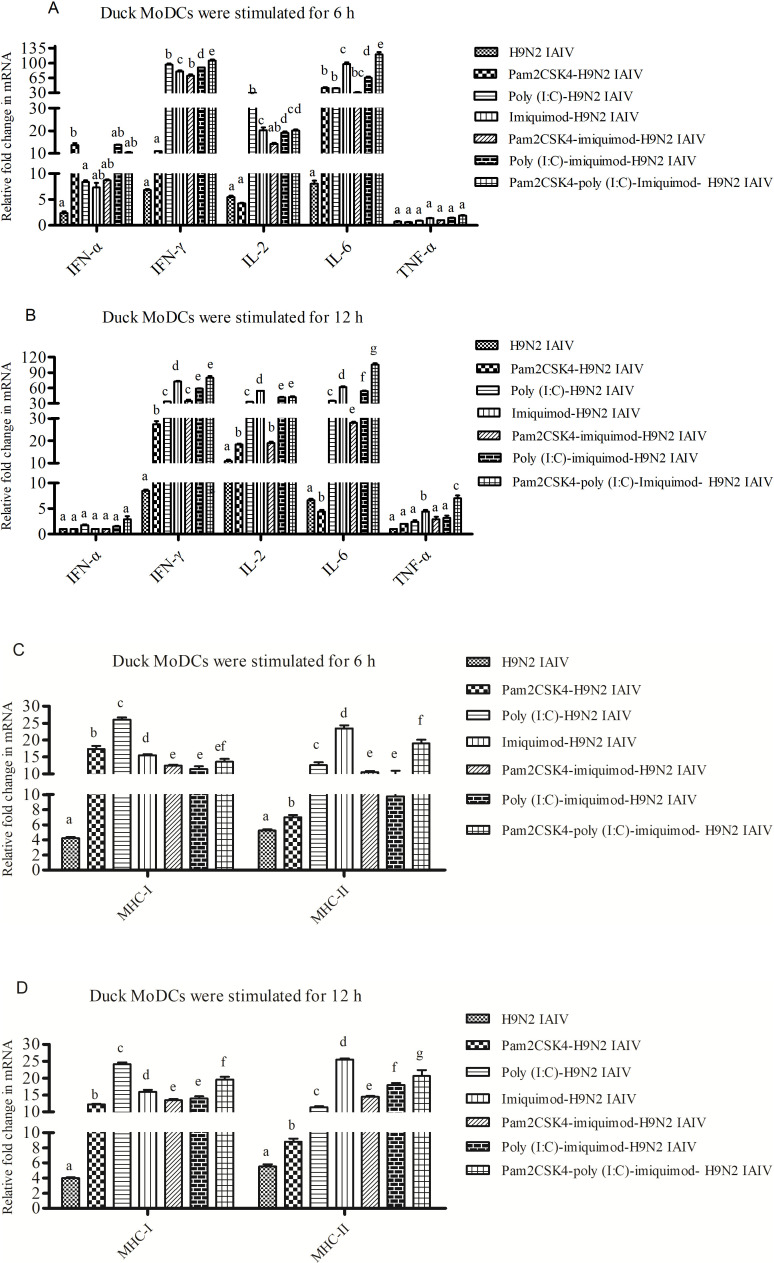
The mRNA levels of Th1/Th2 cytokines and MHC molecules in duck MoDCs stimulated with TLR ligand (s) and H9N2 IAIV at 6 h and 12 h. Then mRNA levels of cytokines (A-B) and MHC molecules (C-D) were detected at 6 h and 12 h post-stimulated via qPCR. Relative mRNA levels are shown as fold changes relative to the level for the control cells of the RPMI-1640 group. Expression of target genes was normalized to those of duck β-Actin. Data were indicated as mean ±SEM. Different letters (a~g) mean significant difference (*P*<0.05). If there is one same letter, for example, ab means has no significant difference between adjacent a or b (*P*>0.05).

*In vivo*, the mRNA expressions of Th1-type cytokines (IL-2 and IFN-γ), Th2-type cytokines (IL-6), proinflammatory cytokines (IFN-α and TNF-α) and cell surface molecules (MHC-I and MHC-II) were up-regulated in almost all TLR ligand(s) -inactivated H9N2 AIV groups compare with inactivated H9N2 AIV group at 6 h post stimulation (Fig 2). The highest relative expression levels of IFN-γ and IL-2 were shown in poly (I:C)-H9N2 IAIV group and pam2CSK4-poly (I:C)-imiquimod-H9N2 IAIV group, respectively. However, IFN-α mRNA showed no statistical difference in all groups at 12 h post stimulation (*P*>0.05). As showed in Fig 2,the relative expression level of TNF-α increased significantly at 12 h post stimulation in imiquimod-H9N2 IAIV group and pam2CSK4-poly(I:C)-imiquimod-H9N2 IAIV group (*P*<0.05). As showed in Fig 2, the highest MHC-I mRNA expression level shown in poly (I:C)-H9N2 IAIV group. However, the highest MHC-II mRNA expression level shown in imiquimod-H9N2 IAIV group (*P*< 0.01).

### Enhances effects of combination of pam2CSK4, poly (I:C) and/or imiquimod on the phagocytosis of H9N2 IAIV in duck MoDCs

**Fig 3:**
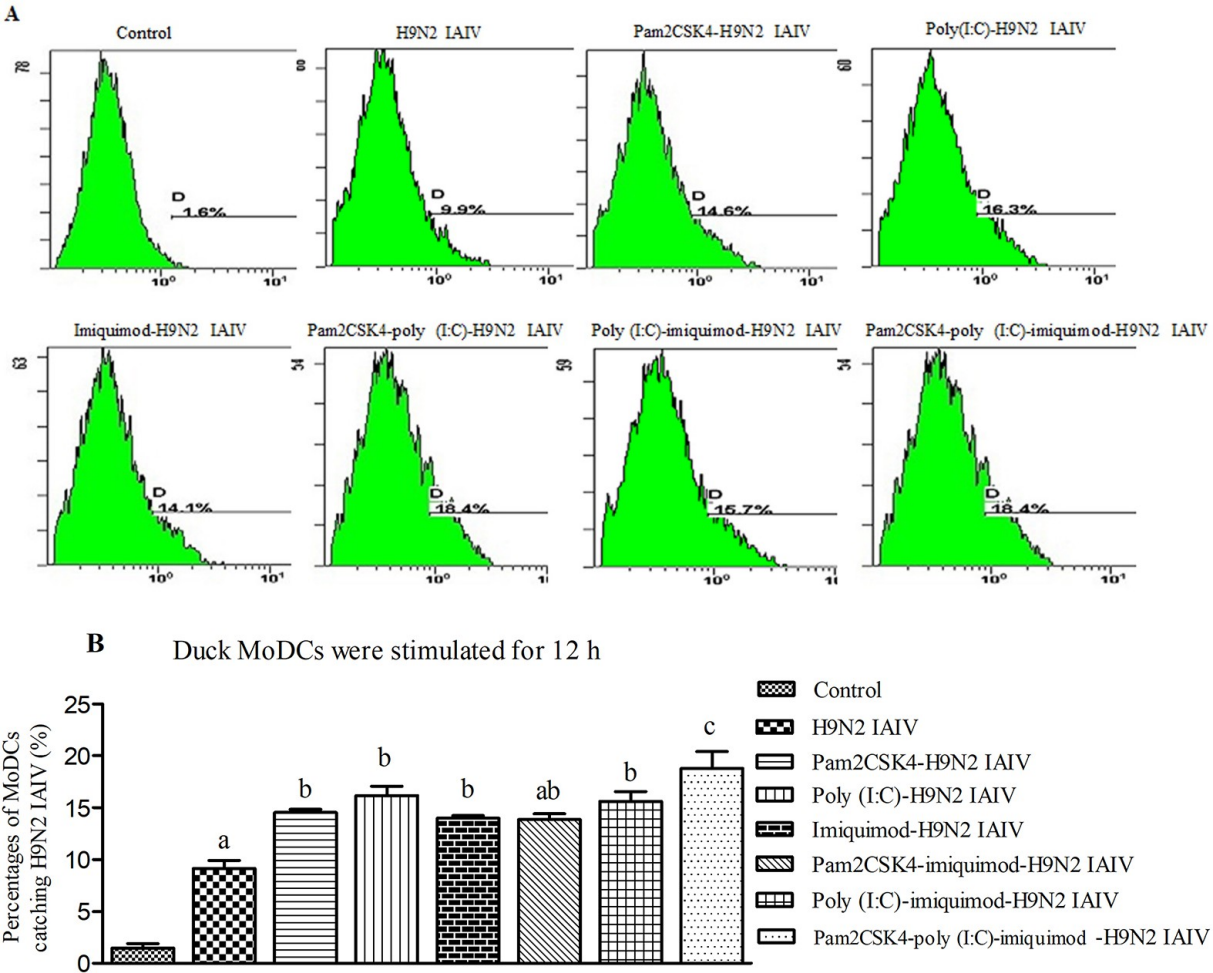
Phagocytosis of MoDCs incubated with pam2CSK4, poly (I:C) and/or imiquimod to H9N2 IAIV antigen at 12 h. (A) Representative flow cytometry profile of the positive rates of MoDCs catching H9N2 IAIV antigen. From group a to group h are RPMI-1640 mock control group, H9N2 IAIV group, pam2CSK4-H9N2 IAIV group, poly (I:C)-H9N2 IAIV group, imiquimod-H9N2 IAIV group, pam2CSK4-imiquimod-H9N2 IAIV group, poly (I:C)-imiquimod-H9N2 IAIV group and pam2CSK4-poly (I:C)-imiquimod-H9N2 IAIV group, respectively. The data presented here are results from one experiment of three flow cytometry experiments. (B) The statistical graph of the positive rates of MoDCs catching H9N2 IAIV antigen. The data were analyzed using Flowjo7.6 software. Data represent the mean ±SEM of three independent experiments. Different letters (a~c) mean significant difference (*P*<0.05); ab means has no significant difference between adjacent a or b (*P*>0.05).

Flow cytometry was conducted to evaluate the capability of MoDCs to phagocytose H9N2 AIV antigen at 12 h post-stimulation. Positive rates of MoDCs catching H9N2 IAIV antigen in RPMI-1640 mock control group, H9N2 IAIV group, pam2CSK4-H9N2 IAIV group, poly (I:C)-H9N2 IAIV group, imiquimod-H9N2 IAIV group, pam2CSK4-imiquimod-H9N2 IAIV group, poly (I:C)-imiquimod-H9N2 IAIV group and pam2CSK4-poly (I:C)-imiquimod-H9N2 IAIV group were 1.5%, 9.1%, 14.5%, 16.2%, 14.1%, 13.8%, 15.6% and 18.9%, respectively (Fig 3). Our results suggested that all TLR ligands (pam2CSK4, poly (I: C) and imiquimod) in our study could enhance the phagocytosis of MoDCs to catch H9N2 IAIV antigen, and the combination of pam2CSK4, poly (I:C) and imiquimod showed the best effect (*P*<0.01).But, there was no statistical difference between combination of the pam2CSK4 and imiquimod to pam2CSK4 or imiquimod alone in the positive rates of MoDCs catching H9N2 IAIV antigen (*P*>0.05).

### Combination of poly (I:C), imiquimod and/or pam2CSK4 adjuvants enhance humoral immune response to H9N2 IAIV in duck

**Fig 4:**
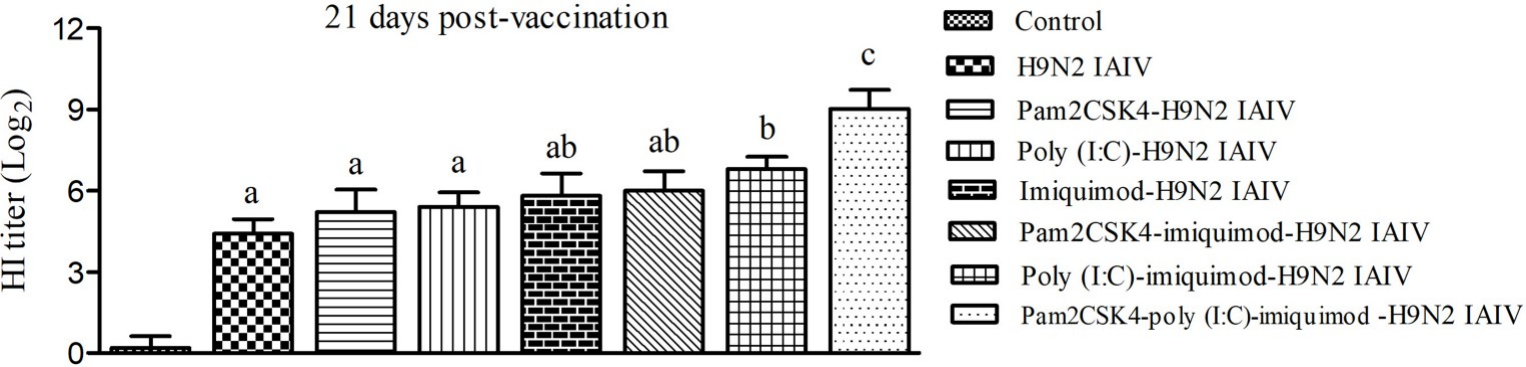
Serum hemagglutination inhibition (HI) levels in duck at 21 dpv.

Humoral immune response was assessed by HI test on serum samples collected at 21dpv. The mean HI titers of RPMI-1640 mock control group, H9N2 IAIV group, pam2CSK4-H9N2 IAIV group, poly (I:C)-H9N2 IAIV group, imiquimod-H9N2 IAIV group, pam2CSK4-imiquimod-H9N2 IAIV group, poly (I:C)-imiquimod-H9N2 IAIV group and pam2CSK4-poly (I:C)-imiquimod-H9N2 IAIV group were 0.2 log2, 4.5 log2, 5.2 log2, 5.4 log2, 5.8 log2, 6 log2, 6.8 log2 and 9 log2, respectively (Fig 4). The HI titers of ducks intraperitoneal immunized with TLR ligand(s)-H9N2 IAIV group (groups A, B, C, D, E and F) showed an insignificant elevation compared with those of the ducks that received only H9N2 IAIV(*P*< 0.05). The highest HI titer was seen in pam2CSK4-poly (I:C)-imiquimod-H9N2 IAIV group (average titer = 9) and which showed significant additive or synergistic effects on HI titers compared to the other group (*P*< 0.01). However, almost no significant difference was observed between the ducks that received imiquimod alone to that received combination of imiquimod and pam2CSK group (*P*>0.05).

Serum samples (5 ducks per group) were collected at 21-day post-vaccination. Antibody titers were determined using the HI assay with 4 HA units of the A/Chicken/Jiangsu/DHN12/2013 H9N2 AIV. The HI titer is expressed as the log2 form. Data were indicated as mean ±SEM. Different letters (a~c) mean significant difference (*P*<0.05); ab means has no significant difference between adjacent a or b (*P*>0.05).

### Combination of poly (I:C), imiquimod and/or pam2CSK4 with H9N2 IAIV enhances cytokines and immuno-related genes producing in duck spleen

**Fig 5:**
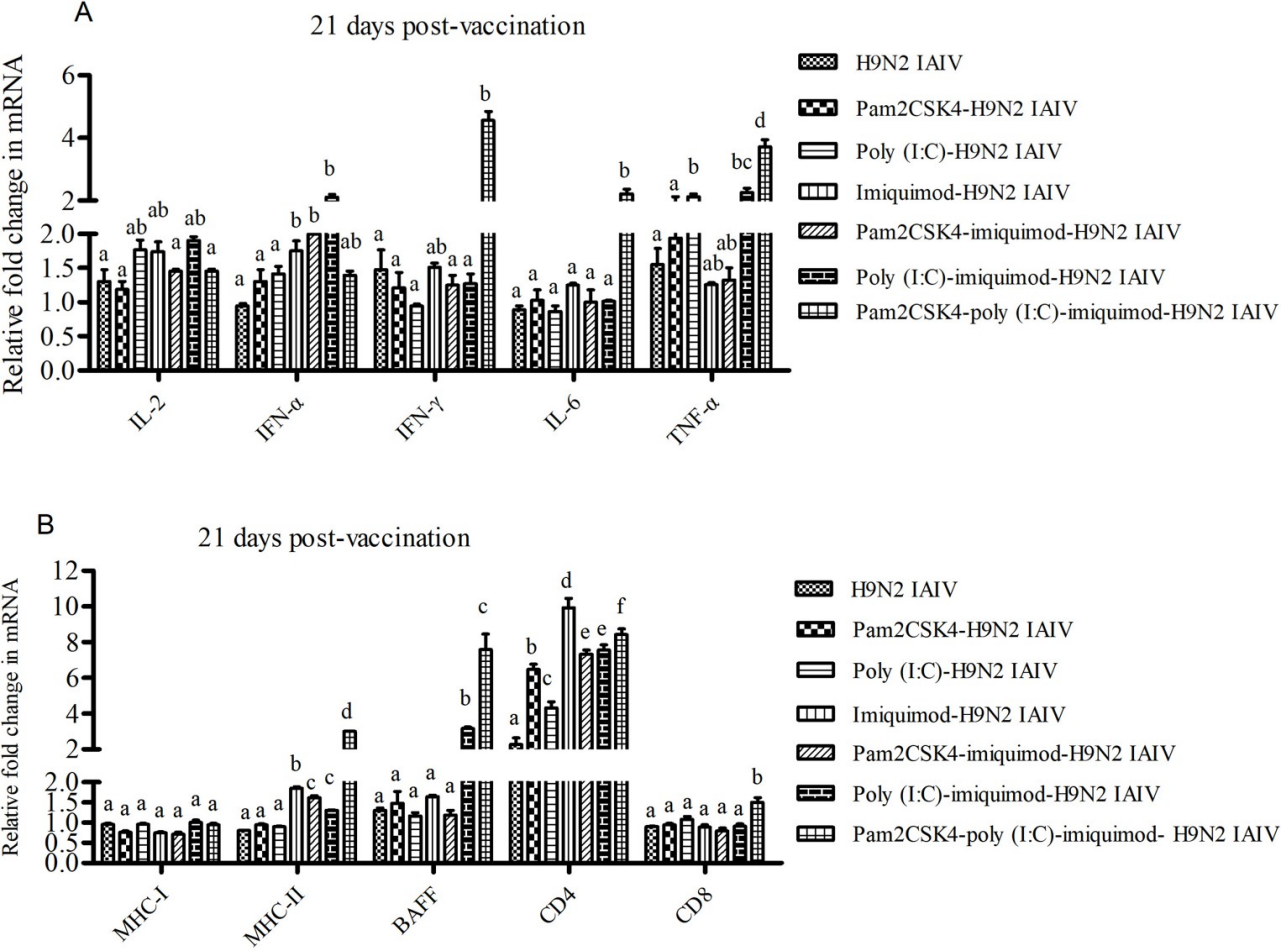
The mRNA levels of cytokines and immuno-related genes in duck spleen at 21dpv. Spleen samples were collected from vaccinated duck at 21 dpv. The mRNA levels of cytokines (IL-2, IFN-α, IFN-γ, IL-6 and TNF-α) (A) and immune-related genes (MHC-I, MHC-II, CD4, CD8 and BAFF) (B) were analyzed by qPCR. Expression of target genes was normalized to those of duck β-Actin. The relative fold change was calculated using PBS injected duck as control. Data were indicated as mean ±SEM. Different letters (a~f) mean significant difference (*P*<0.05). If there is one same letter, for example, ab means has no significant difference between adjacent a or b (*P*>0.05).

To identify potential immunological correlates of effects, gene expression in the spleen of immunized duck was examined at 21 dpv using qPCR. As showed in Fig 5, the highest mRNA relative expression levels of IFN-γ, IL-6, TNF-α and MHC-II mRNA were showed in pam2CSK4-poly (I:C)-imiquimod-H9N2 IAIV group (*P*< 0.05). However, the highest mRNA relative expression levels of IFN-α was showed in poly (I:C)-imiquimod-H9N2 IAIV group (*P*< 0.05).The relative expression level of MHC-II and CD4 mRNA in most TLR ligand(s)-H9N2 IAIV groups (groups C, D, E and F) was significantly up-regulated compare to H9N2 IAIV group (Fig 5). But, the relative expression level of BAFF mRNA was significantly up-regulated only in poly (I:C)-imiquimod-H9N2 IAIV group and pam2CSK4-poly (I:C)-imiquimod-H9N2 IAIV group (Fig 5).

### Combination of poly (I:C), imiquimod and/or pam2CSK4 are effective in activating H9N2 AIV-specific T cells in duck splenic lymphocytes

**Fig 6:**
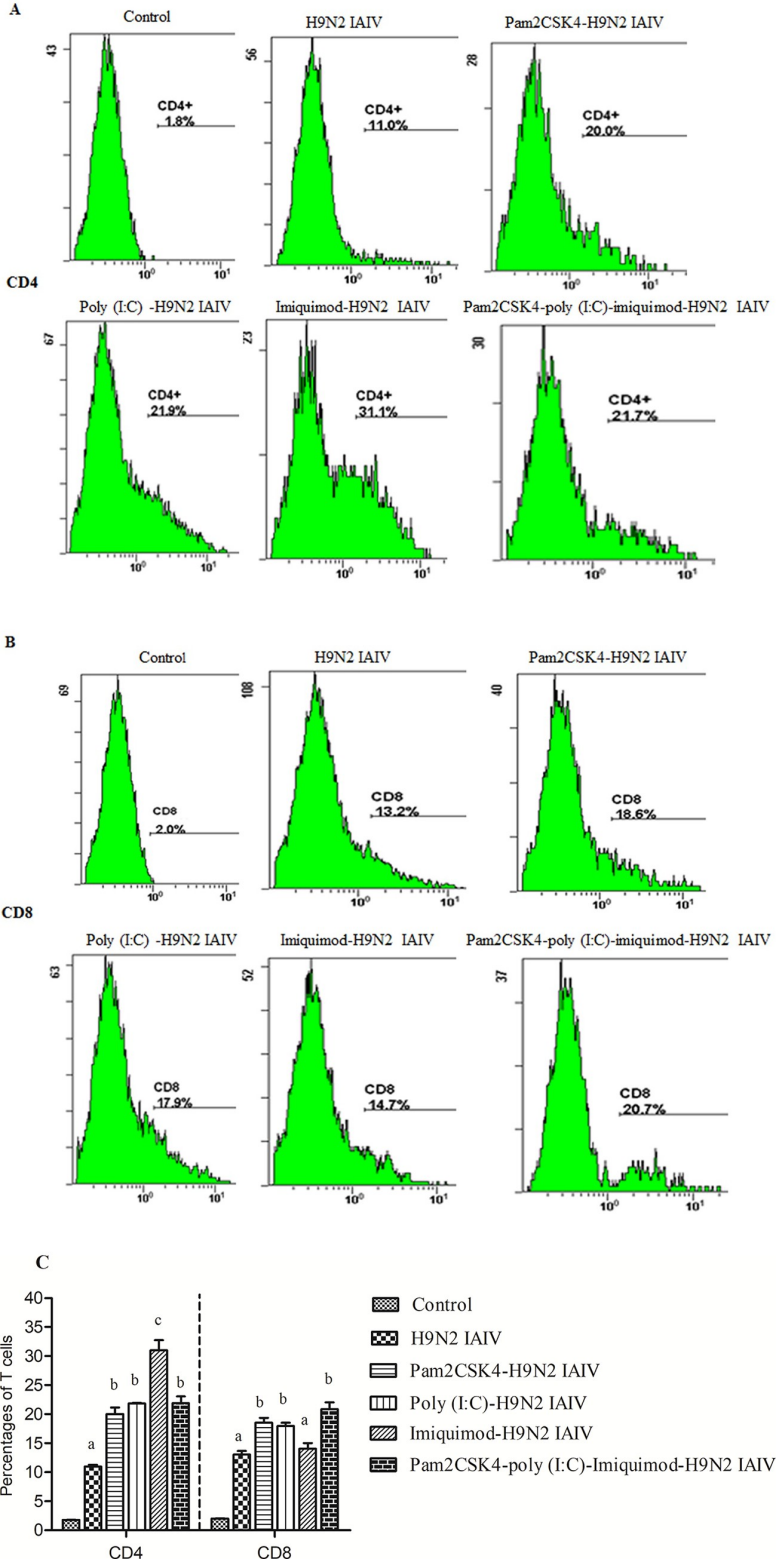
The percentages of CD4^+^ and CD8^+^ T lymphocytes in duck spleen at 21dpv. Splenic lymphocytes were isolated from the spleen of vaccinated duck at 21 dpv. Splenic lymphocyte was subjected to flow cytometry to assess the percentages of CD4^+^ (A) and CD8^+^ (B) T lymphocytes. (C) Data represent the mean ±SEM of three independent experiments. Different letters (a~c) mean significant difference (*P*< 0.05).

Given that the HI titers of pam2CSK4-imiquimod-H9N2 IAIV group and poly (I:C)-imiquimod -H9N2 IAIV group were lower than pam2CSK4-poly (I:C)-imiquimod-H9N2 IAIV group in duck. The percentages of CD4^+^ and CD8^+^ T lymphocytes of duck splenocytes in RPMI-1640 mock control group, H9N2 IAIV group, pam2CSK4-H9N2 IAIV group, poly (I:C)-H9N2 IAIV group, imiquimod-H9N2 IAIV group and pam2CSK4-poly (I:C)-imiquimod-H9N2 IAIV group were analyzed at 21 dpv, the results were 1.8% and 2%, 11% and 13.1%,20% and 18.5%, 21.8% and 18%, 31% and 14%, 21.9% and 20.9%, respectively (Fig 6C). The highest percentages of CD4^+^ and CD8^+^ T lymphocytes were in imiquimod-H9N2 IAIV group and pam2CSK4-poly (I:C)-imiquimod-H9N2 IAIV group, respectively. As the results shown in Fig 6, except for CD8^+^ T lymphocytes in imiquimod-H9N2 IAIV group, percentages of both CD4^+^ and CD8^+^ T lymphocytes in splenocytes of duck immunized with pam2CSK4-H9N2 IAIV, poly (I:C)-H9N2 IAIV, imiquimod-H9N2 IAIV and pam2CSK4-poly(I:C)-imiquimod-H9N2 IAIV was significantly increased when compared with H9N2 AIV antigen alone (*P*< 0.05).

## Discussion

H9N2 AIVs continue to circulate in duck and became the dominant subtype AIVs after 2014, partly reasons of the current inactivated vaccines exhibited lower level of influenza antibody and poor cell mediated immune response in duck when compared to chicken [1,3,5]. Although some studies reported that TLRs ligands as adjuvants for influenza vaccines can enhance immune responses to antigens by activating dendritic cells, relevant data is lacking for H9N2 AIVs in duck [12,14,15,23].

TLR3 signals through the adaptor protein TRIF while TLR2/7 signals through the adaptor protein MyD88, but share some common regulators such as NF-κB [10,11]. The mRNA expression of TRIF /MyD88 and NF-κB in TLR ligand(s)-H9N2 IAIV groups shown up-regulated compare to H9N2 IAIV group at varying degrees, which suggesting TRIF/MyD88-κB signaling pathway has been activated. Also, the highest NF-κB expression level shown in pam2CSK4-poly (I:C)- imiquimod-H9N2 IAIV group which may indicated that this TLR ligands combination inducing the best TLR signaling pathways activation response to H9N2 IAIV in duck MoDCs. It should be noted that due to the lack of antibodies against key node proteins of duck TLR signaling pathway currently, only mRNA expression of related genes was detected in this experiment. However, mRNA expression is a rapid event, so the results of this part need to be further verified in the future.

Cytokines play an important role in the body’s immune response. Poly (I:C) and imiquimod have been demonstrated to induce up-regulation of IFN-α in both birds spleen cells and pig MoDCs [14,15,19]. However, in our study, the mRNA levels of IFN-α of duck MoDCs showed no significantly increased in poly (I:C)-H9N2 IAIV group and imiquimod-H9N2 IAIV group. The reasons may be that partly TLRs ligand induces inconsistent immune effects in different animals or cells, which have been supported by previous studies [23–25]. It is noteworthy that the highest mRNA levels of IFN-γ and IL-6 shown in pam2CSK4-poly (I:C)-imiquimod-H9N2 IAIV group, which suggesting that there is a synergistic effects of pam2CSK4, poly (I:C) and imiquimod on duck MoDCs. Similar to our results, stimulation of murine myeloid dendritic cells with pam3CSK4 and poly (I:C) *in vitro* was shown to increase production of IFN-γ [26].

However, the synergistic effect was not seen in all combination groups. For example, the mRNA levels of cytokines in duck MoDCs of pam2CSK4-imiquimod-H9N2 IAIV group showed no synergistic enhancement effect compares to imiquimod-H9N2 IAIV group. It is could be that TLR ligands combinations with similar signaling pathways may be have a certain thresholds [12,27–29]. Interestingly, positive rates of duck MoDCs catching H9N2 AIV antigen in imiquimod-H9N2 IAIV group are higher than pam2CSK4-imiquimod-H9N2 IAIV group and poly(I:C)-imiquimod-H9N2 IAIV group in present study. The reason may be that maximal responses were achieved by the dose of individual TLR ligands used or higher MHC-II expression in imiquimod-H9N2 IAIV group. From these results, we speculated that single TLR ligand pam3CSK4 or imiquimod inducing weaker activation of TLR signaling pathways in duck MoDCs compare to studies in chicken and mouse, which may due to the species-specific effects.

*In vivo* experiment, the highest levels of IFN-γ, IL-6 and HI titers were showed in pam2CSK4-poly (I:C)-imiquimod-H9N2 IAIV group (*P*< 0.05). It seems that combination of pam2CSK4, poly (I:C) and imiquimod with H9N2 IAIV could induce a mixed Th1-/Th2-like response and given complementary effect on serum titers in duck [30–32]. Also, in our study, the percentages of CD4^+^ and CD8^+^ T lymphocytes of duck splenocytes were higher in pam2CSK4-poly(I:C)-imiquimod-H9N2 IAIV group. Highly expression and production of these proteins in the spleen are crucial for the development of immune responses [30,33–35]. These results suggest that combination of pam2CSK4, poly (I:C) and imiquimod could produce the stronger enhancement immune response to H9N2 IAIV than the other combination of TLR ligands in duck. This synergistic effect is also supported by previous research reports in mice [16,36].

Significantly, HI titers in pam2CSK4-imiquimod-H9N2 IAIV group showed no difference to imiquimod-H9N2 IAIV group, but lower than poly (I:C)-imiquimod- H9N2 IAIV group, indicating that combination of poly (I:C) and imiquimod could produce stronger immune response than combination of pam2CSK4 and imiquimod in duck. Our data showed that both TLR ligands together did not always give any complementary effect on serum titers in all animals. Similarly, vaccination experiments in chickens using an inactivated H9N2 AIV vaccine have shown that combining TLR5 and TLR21 ligands as adjuvants does not lead to synergistic or additive increases in antibody-mediated immune responses compared with administering either ligand alone [23].

Previous works have shown that pam2CSK4 and poly (I:C) could effective enhancing systemic antibody mediated immune responses when administered intramuscularly in chicken and duck when compared with vaccines containing only inactivated influenza virus, respectively [6,16,37]. In the present study, HI titers and the mRNA level of most cytokines in pam2CSK4-H9N2 IAIV group and poly (I:C)-H9N2 IAIV group showed no statistical difference to H9N2 IAIV group. These findings are contrary to previous studies and our *in vitro* experiments, which need further study. It is noteworthy that vaccine administered by intraperitoneal was used in this study, whereas intramuscularly or subcutaneously was used in previously studies. However, previous studies have shown that intraperitoneal imiquimod can augment and accelerate the immune response, and improved vaccine efficacy for H7N9 AIVs in a mouse model [15]. We speculated that intraperitoneal different TLR ligands in different species, may be inducing inconsistent immune responses, and further studies are necessary to explain it in the duck immunity.

## Conclusions

In summary, H9N2 IAIV along with pam2CSK4, poly (I: C) and imiquimod alone or in combination can significantly enhance cell-mediated immune responses and ability of presenting inactivated H9N2 antigen in duck MoDCs, and importantly the interferon effect induced maybe caused by IFN-γ, not IFN-α. Moreover, we have demonstrated that combination of pam3CSK4, poly (I:C) and imiquimod is an effective adjuvant system capable of boosting antibody responses and inducing T cell activation when co-administered with H9N2 IAIV intraperitoneal Muscovy duck. It is also shown that combining pam2CSK4 with imiquimod compare to imiquimod alone did not have significant additive and synergistic effects on antibody responses of H9N2 IAIV in Muscovy duck. Nevertheless, future experiments should examine the immunity protective efficacy to H9N2 AIVs with different combinations of TLR ligands vaccine by different vaccination strategies in duck to further verify the conclusions of this paper.

## Supporting information

S1 Fig Morphological characteristics of duck MoDCs. (TIF)

S2 Fig Detection of MHC-II expression on duck MoDCs by flow cytometry. (TIF)
