# Supplementary figures and images for: Evaluation of different combination of pam2CSK4, poly (I:C) and imiquimod enhance immune responses to H9N2 avian influenza antigen in dendritic cells and duck
Source: PLoS One. 2022 Jul 19;17(7):e0271746. doi: 10.1371/journal.pone.0271746 (PMC9295992; doi:10.1371/journal.pone.0271746)

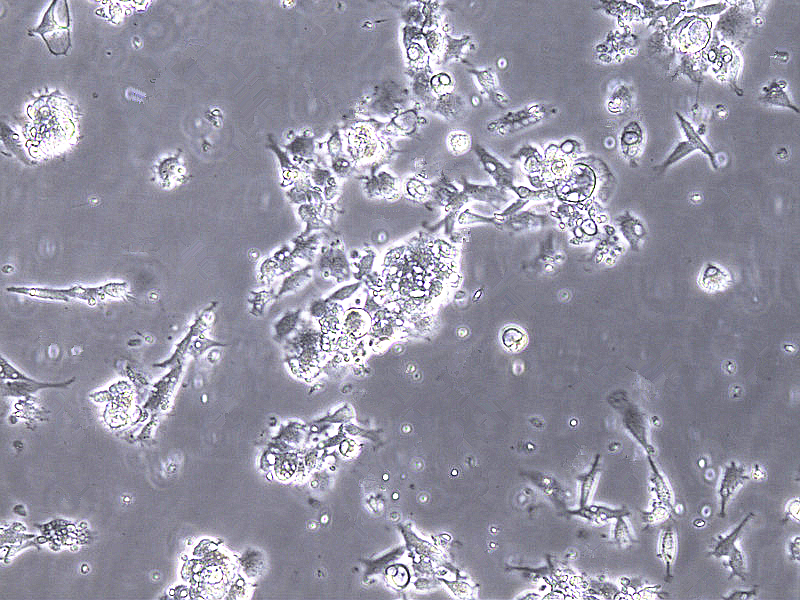

Supplement: S1 Fig — (TIF) [file pone.0271746.s001.tif]

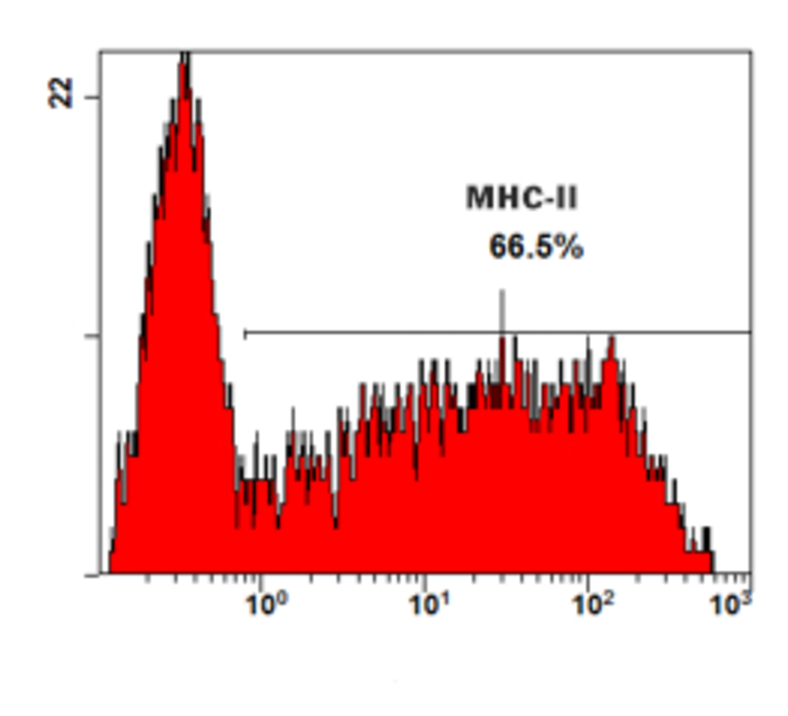

Supplement: S2 Fig — (TIF) [file pone.0271746.s002.tif]
